# MYC in Oncogenesis and Therapeutic Implications

**DOI:** 10.1002/mco2.70893

**Published:** 2026-07-27

**Authors:** Na Zhang, Li Wei, Cuiping Tang, Peilong Lai, Wenbin Zhong, Hua You

**Affiliations:** ^1^ Department of Pediatric Oncology Sichuan Clinical Research Center for Cancer Sichuan Cancer Hospital & Institute Sichuan Cancer Center Affiliated Cancer Hospital of University of Electronic Science and Technology of China Chengdu China; ^2^ Chongqing Health Center for Women and Children Women and Children's Hospital of Chongqing Medical University NHC Key Laboratory of Birth Defects and Reproductive Health Chongqing China; ^3^ Department of Hematology Guangdong Provincial People's Hospital Guangdong Academy of Medical Sciences Guangzhou China; ^4^ Guangzhou Institute of Cancer Research The Affiliated Cancer Hospital Guangzhou Medical University Guangzhou China

**Keywords:** clinical trials, indirect targeting, mechanism, myc oncogene, therapeutic targets

## Abstract

The MYC oncogene family constitutes a master regulatory hub in tumorigenesis, with functional complexity extending far beyond individual gene activities. Recent advances unveil cooperative yet context‐dependent antagonism and dynamic interplay among MYC family members, fundamentally reshaping our understanding of lineage specific oncogenic programs. This review synthesizes emerging insights into MYC orchestrated tumor microenvironment remodeling, reciprocal regulation with noncoding RNAs, and the transcriptional and epigenetic governance of metabolic reprogramming. We delineate mechanisms by which MYC drives therapeutic resistance and critically evaluate current strategies targeting MYC or its downstream networks, encompassing direct MYC–MAX disruptors, upstream pathway inhibitors, synthetic lethality, and combinatorial regimens with immune checkpoint blockade or conventional chemotherapy. We further discuss MYC's prognostic significance across diverse cancer types, the critical gap between preclinical efficacy and clinical outcomes, and emerging combination strategies aimed at overcoming acquired drug resistance. By integrating these rapidly evolving biological dimensions, we posit MYC as a highly multidimensional regulatory node whose context‐dependent functions present both formidable challenges and promising new opportunities for effective therapeutic intervention.

## Graphical abstract

The MYC oncogene family (c‐MYC, N‐MYC, L‐MYC) functions as a central transcriptional hub orchestrating cancer progression through multiple interconnected pathways. This graphical abstract illustrates MYC's role as a multidimensional regulator positioned at the nexus of tumor microenvironment remodeling, metabolic reprogramming, and therapeutic resistance. MYC drives comprehensive tumor microenvironment transformation through immune evasion mechanisms (PD‐L1 upregulation, antigen presentation suppression), vascular plasticity (VEGF‐mediated angiogenesis), and fibroblast activation. Simultaneously, MYC orchestrates metabolic reprogramming via enhanced glycolysis, glutaminolysis, and lipid biosynthesis, creating nutrient‐competitive tumor ecosystems. Reciprocal regulation with noncoding RNAs (miRNAs, lncRNAs, circRNAs) forms complex feedback loops modulating MYC stability and oncogenic output. These multifaceted functions contribute to therapeutic resistance across multiple treatment modalities. Targeting strategies include direct MYC–MAX inhibitors (Omomyc), BET bromodomain inhibitors (JQ1), and metabolic targeting approaches, highlighting both challenges and opportunities for precision oncology in MYC‐driven malignancies.

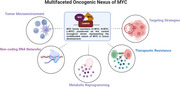

## 1 Introduction

The MYC oncogene family includes c‐MYC, N‐MYC, and L‐MYC, which encodes transcription factors that govern a wide range of cellular processes, including proliferation, differentiation, apoptosis, and metabolism [1]. Deregulation of MYC family members is a hallmark of many human cancers, where their aberrant activation drives tumor initiation and progression across diverse tissue types [2]. Mounting evidence now indicates that MYC proteins do not function in isolation but engage in dynamic, lineage‐specific crosstalk through cooperative and antagonistic interactions, challenging the traditional gene‐centric view of MYC biology [3, 4]. These oncogenic programs are deeply embedded in cellular context and shaped by multifaceted regulatory networks, including chromatin remodeling, noncoding RNA (ncRNA) interactions [5], and modulation of the tumor microenvironment (TME) [6]. MYC further orchestrates metabolic reprogramming by regulating nutrient uptake, mitochondrial dynamics, and biosynthetic flux, underscoring its role as a master regulator of cancer cell fitness. Critically, MYC drives therapeutic resistance through epigenetic plasticity and bypass signaling activation [7, 8].

Given the pervasive influence of MYC across oncogenic processes, its direct targeting has long been considered a “holy grail” in cancer therapy [9, 10]. Nevertheless, the inherent structural disorder of MYC proteins and their essential physiological roles have hindered conventional drug development. Recent advancements, including targeted protein degradation, synthetic lethality, epigenetic modulation, and combinatorial approaches with immunotherapy, are now being actively investigated in preclinical and early clinical studies. These developments underscore the translational potential of MYC‐targeted interventions while highlighting the need for a critical synthesis of current knowledge [11].

In this review, we integrate contemporary insights into MYC's multifaceted roles in oncogenesis. First, we analyze the cooperative and antagonistic dynamics among MYC family members and their lineage‐specific functions. We then explore MYC's dual influence on TME remodeling, ncRNA regulation, and metabolic reprogramming. Critically, we evaluate MYC's contribution to therapeutic resistance and highlight emerging therapeutic strategies including both direct MYC inhibitors and indirect targeting of upstream regulatory pathways (YAP/TAZ, p53/MDM2, PI3K/AKT/mTOR, WNT/β‐catenin, BRD4/BET, CDK7/9) [12, 13, 14]. By synthesizing these interconnected aspects, we position MYC not merely as a classical oncogenic driver but as a context‐dependent biological orchestrator whose multidimensional functions present both challenges and actionable opportunities for precision oncology.

## 2 Domain Structures and Multifaceted Roles of MYC in Cancer

The structural features and functional modularity of MYC paralogs provide the foundation for understanding their diverse oncogenic roles. The MYC gene family encodes three basic helix‐loop‐helix leucine zipper (bHLH‐LZ) transcription factors: c‐MYC, N‐MYC, and L‐MYC [15]. They share conserved domains including the bHLH‐LZ DAN‐binding motif and MYC boxes (MB) yet display distinct expression patterns, regulatory mechanisms, and tissue‐specific oncogenic activities [16]. While extensive studies have focused on the tumorigenic roles of MYC protein, mounting evidence suggests that functional interplay among family members may critically shape the oncogenic landscape [17].

### 2.1 Physiological Roles of MYC in Normal Cells

Under physiological conditions, MYC family transcription factors integrate mitogenic, metabolic, and developmental cues as master regulatory hubs [18]. These paralogs share the conserved bHLH‐LZ architecture, enabling obligatory heterodimerization with MAX and recognition of canonical E‐box motifs (5’‐CACGTG‐3’) within target gene regulatory regions. Through this platform, MYC proteins coordinate ribosomal biogenesis, metabolism, mitochondrial dynamics, and cell cycle progression, balancing proliferative capacity with terminal differentiation [19]. In healthy tissues, MYC expression is subject to tight spatiotemporal control, with low basal levels and transient induction in response to growth signals [20, 21]. Each paralog exhibits distinct tissue distribution: c‐MYC is ubiquitously expressed, N‐MYC is predominantly restricted to neural and neuroendocrine tissues, and L‐MYC is enriched in pulmonary neuroendocrine cells and lymphoid subsets. This differential expression underpins nonredundant developmental functions [22]. The physiological relevance of MYC is further underscored by its extreme dosage sensitivity. Haploinsufficiency of MYC or its paralogs causes embryonic lethality or severe developmental defects, whereas somatic overexpression predisposes to oncogenic transformation [23].This narrow window between physiological necessity and pathological excess highlights the critical importance of precise MYC regulation in maintaining tissue homeostasis [20].

### 2.2 Domain Structures and Functional Modularity of MYC Family Members

**FIGURE 1:**
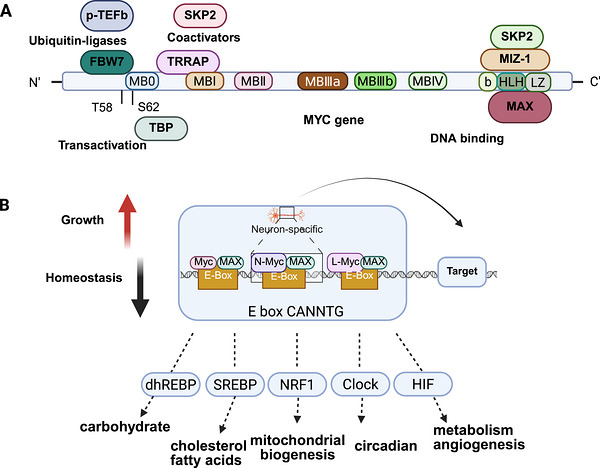
Domain organization of MYC and its integration into growth‐ and homeostasis‐related transcriptional networks. (A) Schematic diagram of MYC domain organization, showing the N‐terminal transactivation region containing conserved MYC boxes and the C‐terminal basic helix‐loop‐helix leucine zipper (bHLH‐LZ) domain required for MYC‐associated factor X (MAX) dimerization and E‐box binding. Representative MYC‐interacting cofactors and regulatory proteins, as well as the major phosphorylation sites threonine 58 (T58) and serine 62 (S62), are indicated. (B) MYC/MAX heterodimers bind E‐box elements in target genes and cooperate with context‐dependent transcriptional regulators to control diverse biological programs related to growth and homeostasis, including carbohydrate metabolism, cholesterol and fatty acid metabolism, mitochondrial biogenesis, circadian regulation, and hypoxia‐related metabolic adaptation.

The MYC oncogene family comprises c‐MYC (located at 8q24), N‐MYC (located at 2p24), and L‐MYC (located at 1p34), which encode bHLH‐LZ transcription factors belonging to the basic bHLH‐LZ superfamily of transcription factors [24]. This conserved domain is essential for transcriptional activation of target genes involved in proliferation, metabolism, and biosynthesis. Significant divergence exists in their N‐terminal TADs, which contain paralog‐specific motifs and distinct regulatory elements for posttranslational modifications (PTMs) [22, 25]. All three proteins contain multiple highly conserved regions known as MB, which are critical for their function. c‐MYC and N‐MYC each contain six MB (MB0, MBI, MBII, MBIIIa, MBIIIb, and MBIV), whereas L‐MYC lacks MBIII. MB0, MBI, and MBII are located within the TAD of the MYC proteins, while the remaining MBs are situated in the central region of the protein [26] (Figure 1A). These complexes enable chromatin opening and transcriptional elongation. In contrast, N‐MYC and L‐MYC display modifications or absence of certain MB, altering their transcriptional potency and co‐regulator engagement [27]. Each MYC protein recruits a distinct set of interacting proteins and exerts divergent functions within the cell (Figure 1B). For instance, N‐MYC interacts preferentially with aurora kinase A (AURKA) in neuroblastoma, which stabilizes N‐MYC by preventing its ubiquitin‐mediated degradation [28]. L‐MYC acts as a weaker transactivator and associates with factors involved in pulmonary neuroendocrine cell differentiation, potentially driving lineage‐specific transcriptional programs in small cell lung cancer (SCLC) [29]. PTMs further diversify the stability and activity profiles among MYC paralogs [30]. For example, phosphorylation of c‐MYC at Thr58 targets it for FBXW7‐mediated degradation, whereas N‐MYC is stabilized by PI3K‐AKT dependent phosphorylation at Ser62 [31, 32]. These differences in domain architecture and PTM susceptibility impart paralog‐specific transcriptional dynamics and cofactor preferences.

### 2.3 Tissue‐Specific Oncogenic Programs of c‐MYC, N‐MYC, and L‐MYC

Although MYC paralogs share overlapping DNA‐binding specificities, their oncogenic activities are highly context‐dependent, arising from lineage‐restricted expression patterns, chromatin accessibility, and integration with developmental signaling pathways [33]. c‐MYC exhibits broad tissue distribution and drives tumorigenesis across multiple malignancies, including colorectal, breast, lung, and hematological cancers [34]. In colorectal cancer, it functions as a downstream effector of WNT/β‐catenin signaling, driving stem cell expansion; in Burkitt lymphoma, chromosomal translocation t(8;14) leads to constitutive c‐MYC activation and B‐cell hyperproliferation [35, 36]. N‐MYC displays more restricted expression, predominantly in neural and neuroendocrine tissues. It associates with super‐enhancers regulating cell cycle and mitotic genes and critically depends on AURKA for stabilization. N‐MYC amplification drives aggressive neuroblastoma and has been linked to extrachromosomal DNA (ecDNA)–mediated gene dosage effects [23]. L‐MYC functions as a weaker transcriptional activator with a narrower oncogenic spectrum, selectively expressed in neuroendocrine tumors, particularly SCLC [37, 38].

Critically, MYC functions as a molecular rheostat for lineage identity, as shown in Table 1. In SCLC, c‐MYC activation in L‐MYC‐driven neuroendocrine cells induces a non‐neuroendocrine variant phenotype through EZH2‐mediated chromatin remodeling [39]. This MYC‐dependent plasticity represents a therapeutic vulnerability, as variant SCLC exhibits enhanced sensitivity to Aurora kinase inhibition but acquired resistance to platinum‐based chemotherapy. Similarly, in prostate cancer, MYC amplification drives lineage plasticity from adenocarcinoma to neuroendocrine differentiation through cooperative interactions with epigenetic regulators EZH2 and SOX2 [40]. This paralog‐selective deployment reflects evolutionary subfunctionalization, demanding lineage‐aware diagnostic frameworks and paralog‐directed therapeutic strategies.

**TABLE 1:** Systematic comparison of MYC family members.

Feature	c‐MYC	N‐MYC	L‐MYC
Expression	Ubiquitous	Neural/neuroendocrine	Neuroendocrine
Major cancers	CRC, breast, lymphoma	Neuroblastoma, medulloblastoma	SCLC
Genomic alteration	Translocation, amplification	Amplification	Amplification
Key dependency	WNT signaling	AURKA stabilization	Lineage TFs
Transcriptional strength	Strong	Strong	Weak
Clinical relevance	Broad	High‐risk marker	Subtype‐specific

### 2.4 Cooperative and Antagonistic MYC Regulation in Tumorigenesis

Evidence for true functional nonredundancy versus context‐dependent expression among MYC paralogs remains debated. Recent lineage‐tracing studies suggest that apparent antagonism may reflect cell‐of‐origin effects rather than intrinsic biochemical differences [41]. In SCLC, c‐MYC, N‐MYC, and L‐MYC define predominantly mutually exclusive molecular subtypes: c‐MYC‐driven subtypes exhibit hyperproliferative signaling and increased chromatin accessibility; L‐MYC is enriched in neuroendocrine subtypes sustaining expression of achaete‐scute homolog 1 (ASCL1) and neuronal differentiation 1 (NEUROD1); and N‐MYC amplification drives pediatric cancers such as neuroblastoma and medulloblastoma by promoting undifferentiated, stem‐like states [42]. These observations establish that MYC paralogs have evolved specialized functions dictated by developmental lineage and epigenetic context. MYC family members also engage in antagonistic interactions that alter cellular identity. In SCLC, c‐MYC suppresses neuroendocrine markers and induces phenotype switching, promoting lineage plasticity. Mechanistically, such antagonism arises from differential cofactor recruitments. c‐MYC and L‐MYC activate divergent gene sets through distinct interactions with transformation/transcription domain‐associated protein (TRRAP), WD repeat domain 5 (WDR5), and histone acetyltransferase p300, influencing enhancer selection and chromatin accessibility [43, 44, 45]. Competition for shared dimerization partners like MAX further alters complex composition. This dynamic antagonism facilitates therapy resistance through phenotype switching. Conversely, MYC paralogs can synergize under oncogenic conditions [46]. In tumors with dysregulated MAX or MGA, multiple family members cooperate to sustain proliferation and metabolic rewiring through shared enhancer networks and coactivator complexes including SWI/SNF and Mediator [47]. This cooperativity is highly plastic and capable of rewiring under therapeutic stress [48, 49]. Importantly, this creates synthetic vulnerabilities: N‐MYC‐amplified neuroblastoma cells show sensitivity to cyclin‐dependent kinase 7 (CDK7)/CDK9 or bromodomain and extraterminal domain (BET) inhibitors, and disrupting cofactors such as bromodomain‐containing protein 4 (BRD4) or transcription factor II H (TFIIH) can induce synthetic lethality in tumors codependent on multiple MYC paralogs. Targeting dynamic paralog interactions, rather than individual proteins, thus represents a paradigm‐shifting strategy [50, 51].

### 2.5 Clinical and Therapeutic Implications of MYC Family Dynamics

The dynamic behavior of MYC family members has profound implications for clinical management and therapeutic targeting [52]. Paralog‐specific dependencies have emerged as notable vulnerabilities. N‐MYC amplification is well‐established as a poor prognostic indicator in neuroblastoma and serves as a critical component in risk stratification protocols [53]. In medulloblastoma, high c‐MYC/N‐MYC expression defines Group 3 subtypes, correlating with poor survival and informing molecular classification systems. Targeted therapies exploiting MYC paralog dependencies are under active development. In N‐MYC amplified neuroblastoma, AURKA inhibitors such as alisertib promote N‐MYC degradation by disrupting the AURKA‐N‐MYC stabilization complex [54]. Early‐phase trials have demonstrated efficacy in combination with chemotherapy, although toxicity and resistance remain concerns [55, 56, 57]. Resistance via compensatory c‐MYC upregulation has been observed, suggesting that tumors shift paralog dependency to escape therapeutic pressure. This inter‐paralog compensation underscores the need for strategies that counteract paralog switching. Agents targeting shared MYC transcriptional machinery offer promising alternatives. BRD4 and CDK7/12/13 inhibitors disrupt core MYC‐dependent transcription irrespective of paralog identity and are under clinical investigation [58]. Direct MYC–MAX dimerization inhibitors, including OmoMYC and MYCi975, have shown potent antitumor activity in preclinical studies; OmoMYC has progressed to Phase I trials in advanced solid tumors. However, challenges related to delivery, bioavailability, and on‐target toxicity in normal tissues remain [59, 60, 61].

Clinical implementation of MYC‐targeted strategies requires robust biomarkers for real‐time monitoring. Single‐cell RNA sequencing and spatial transcriptomics now enable high‐resolution mapping of MYC paralog expression across tumor regions. Circulating tumor DNA (ctDNA) and cell‐free RNA analysis offer minimally invasive methods to monitor MYC amplification status and treatment‐induced reprogramming [18]. In summary, MYC paralogs exert nonredundant, context‐specific functions; inter‐paralog switching represents a clinically relevant resistance mechanism; and effective therapies will likely require real‐time molecular monitoring and combination‐based treatment designs. Understanding MYC family dynamics may enable rational design of context‐adapted regimens for patients with MYC‐driven cancers.

## 3 The Role of MYC in Shaping the TME

Beyond its well‐established role in intrinsic tumor cell proliferation, MYC orchestrates profound remodeling of the TME through transcriptional, metabolic, and paracrine programs [62]. As a master regulator, MYC reprograms immune surveillance, angiogenic signaling, stromal interactions, and metabolic competition functions essential for sustaining tumor progression and mediating resistance to therapy, particularly immunotherapy [63].

### 3.1 Immune Evasion and Immunosuppressive Reprogramming

**FIGURE 2:**
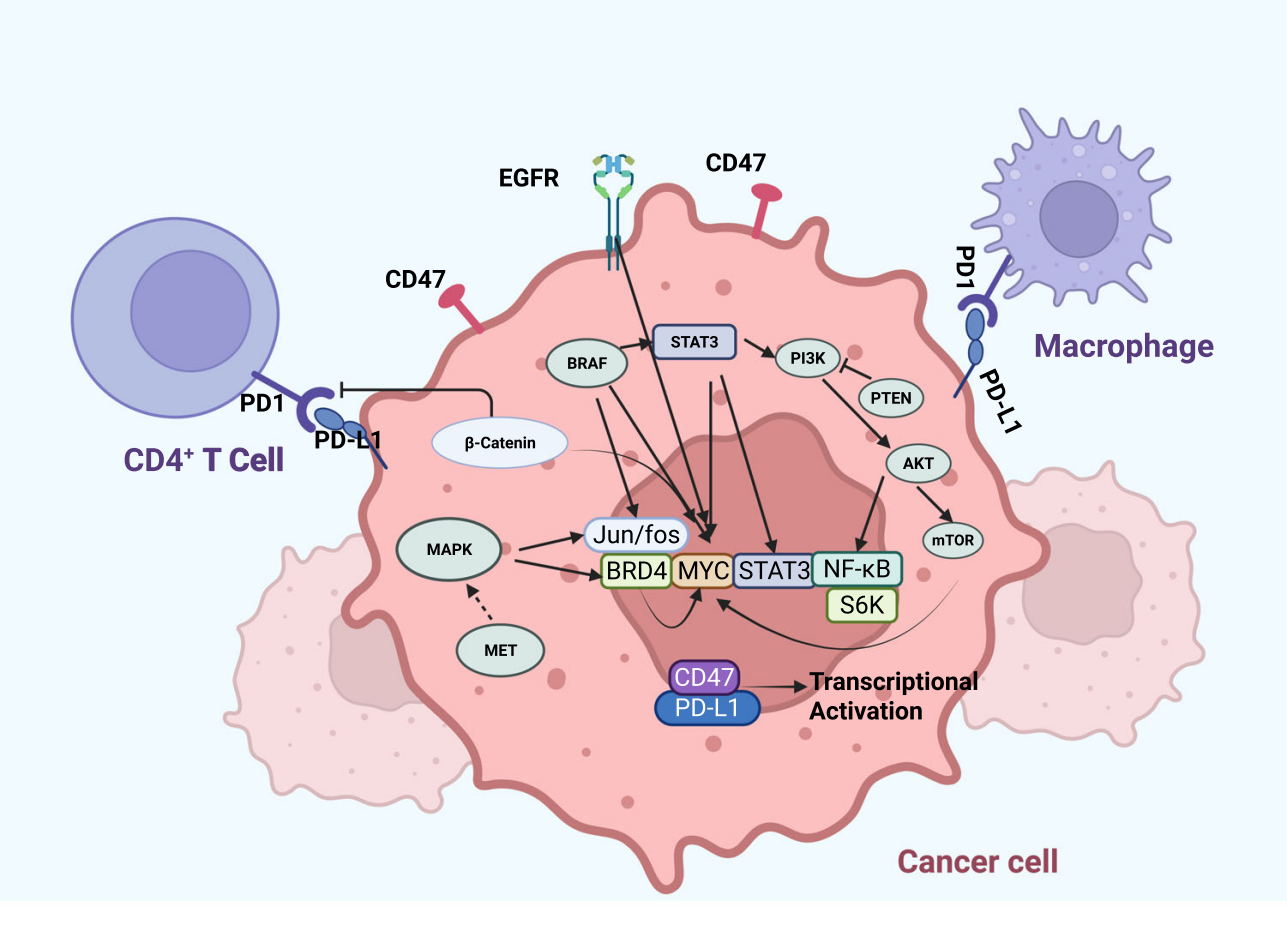
The MYC oncoprotein orchestrates transcriptional programs critical for tumor growth and immune evasion. MYC acts as a central node converging epidermal growth factor receptor‐signal transducer and activator of transcription 3 (EGFR‐STAT3), B‐Raf proto‐oncogene (BRAF)‐mitogen activated protein kinase (MAPK), mesenchymal–epithelial transition factor (MET), and Wingless‐related integration site (Wnt)/β‐catenin/bromodomain‐containing protein 4 (BRD4) signaling to activate transcription of proliferation genes. MYC also transcriptionally upregulates programmed death‐ligand 1 (PD‐L1) and cluster of differentiation 47 (CD47), which engage programmed death‐1 (PD‐1) on CD4^+^ T cells and signal regulatory protein alpha (SIRPα) on macrophages, respectively, thereby suppressing antitumor immunity through T cell inhibition and phagocytosis evasion.

MYC orchestrates immune evasion through direct transcriptional regulation and indirect cellular network reprogramming, enabling malignant cells to evade immune surveillance and resist immunotherapy [64] (Figure 2). While most insights have focused on c‐MYC, emerging data suggest that N‐MYC and L‐MYC also contribute in paralog‐ and context‐specific manners [63]. c‐MYC binds directly to the CD274 promoter to drive PD‐L1 expression in lung, breast, and colorectal cancers, dampening cytotoxic T cell activity [65]. Concurrently, it represses MHC Class I components and peptide transporters (TAP1, TAP2), reducing tumor immunogenicity [66]. This dual action upregulating inhibitory checkpoints while silencing antigen visibility allows tumor cells to persist within immunocompetent hosts. The relative contribution of these two mechanisms likely varies across tumor types, with implications for patient stratification in combination therapies [67]. Beyond tumor‐intrinsic effects, c‐MYC remodels the immune landscape through cytokine secretion like CCL2, CSF1, and transforming growth factor‐β (TGF‐β), which recruits immunosuppressive subsets. MYC‐driven metabolic remodeling further depletes glucose, tryptophan, and arginine while inducing indoleamine 2,3‐dioxygenase 1 (IDO1) and arginase 1 (ARG1) expression, contributing to T cell exhaustion. Whether this metabolic immunosuppression represents a direct transcriptional program or an emergent consequence of proliferative demand remains unresolved, with significant implications for therapeutic targeting [68, 69].

N‐MYC amplified neuroblastomas exhibit low MHC‐I and poor T cell infiltration, producing an immune‐desert phenotype resistant to checkpoint blockade [70]. Whether N‐MYC directly represses antigen presentation or acts indirectly through differentiation programs remains debated. L‐MYC, expressed in SCLC neuroendocrine subtypes, is associated with low immunogenicity; notably, c‐MYC‐driven SCLC subtypes show greater immunogenicity and better checkpoint inhibitor responsiveness, suggesting functional divergence between c‐MYC and L‐MYC [71, 72]. However, most studies rely on cell line models that may not recapitulate spatial immune exclusion in vivo, and the translational feasibility of combining MYC‐targeted therapies with immunotherapy requires clinical validation [73, 74].

### 3.2 Vascular Plasticity and Neo‐Angiogenesis

MYC proteins, c‐MYC and N‐MYC particularly, are key regulators of tumor angiogenesis through direct transcriptional control and endothelial signaling pathway interactions [75, 76]. Rather than merely inducing generic pro‐angiogenic programs, MYC drives formation of vasculature characterized by structural abnormalities and immunosuppressive functionality [77]. c‐MYC directly upregulates VEGFA by binding to its promoter and enhancing enhancer accessibility, while also activating ANGPT2 and PDGFB and suppressing angiostatic THBS1 [78, 79]. While VEGF upregulation is shared across oncogenic pathways, c‐MYC's simultaneous THBS1 suppression and promotion of endothelial‐to‐mesenchymal transition (EndMT) represent distinctive, MYC specific angiogenic programs [41]. Endothelial‐specific c‐MYC deletion disrupts tumor angiogenesis and restores vessel morphology in mouse models. N‐MYC enhances VEGF and fibroblast growth factor receptor (FGFR1) expression, promoting endothelial recruitment [80]. N‐MYC driven tumors frequently display chaotic vasculature and hypoxia that reinforce N‐MYC stability via HIF‐1α feedback loops, which represents a potentially targetable vulnerability, as dual N‐MYC/VEGF inhibition reduces vascular density in preclinical models [81, 82]. L‐MYC's angiogenic role remains unexplored, though preliminary data suggest indirect effects via neuroendocrine differentiation programs [82, 83]. MYC also regulates vascular plasticity, enabling dynamic remodeling under hypoxia or therapeutic stress [84]. Tumor endothelial MYC signaling downregulates adhesion molecules ICAM1 and VCAM1, thereby compromising leukocyte tethering and transmigration and erecting a physical barrier to T cell entry [85, 86]. Such MYC‐dependent vascular reprogramming sets these tumors apart from other angiogenic cancers and likely explains why anti‐VEGF therapy alone rarely achieves meaningful T cell infiltration [87]. Thus, MYC functions as a molecular bridge linking vascular pathology and immune evasion.

### 3.3 Fibroblast Phenotypes and Matrix Remodeling

**FIGURE 3:**
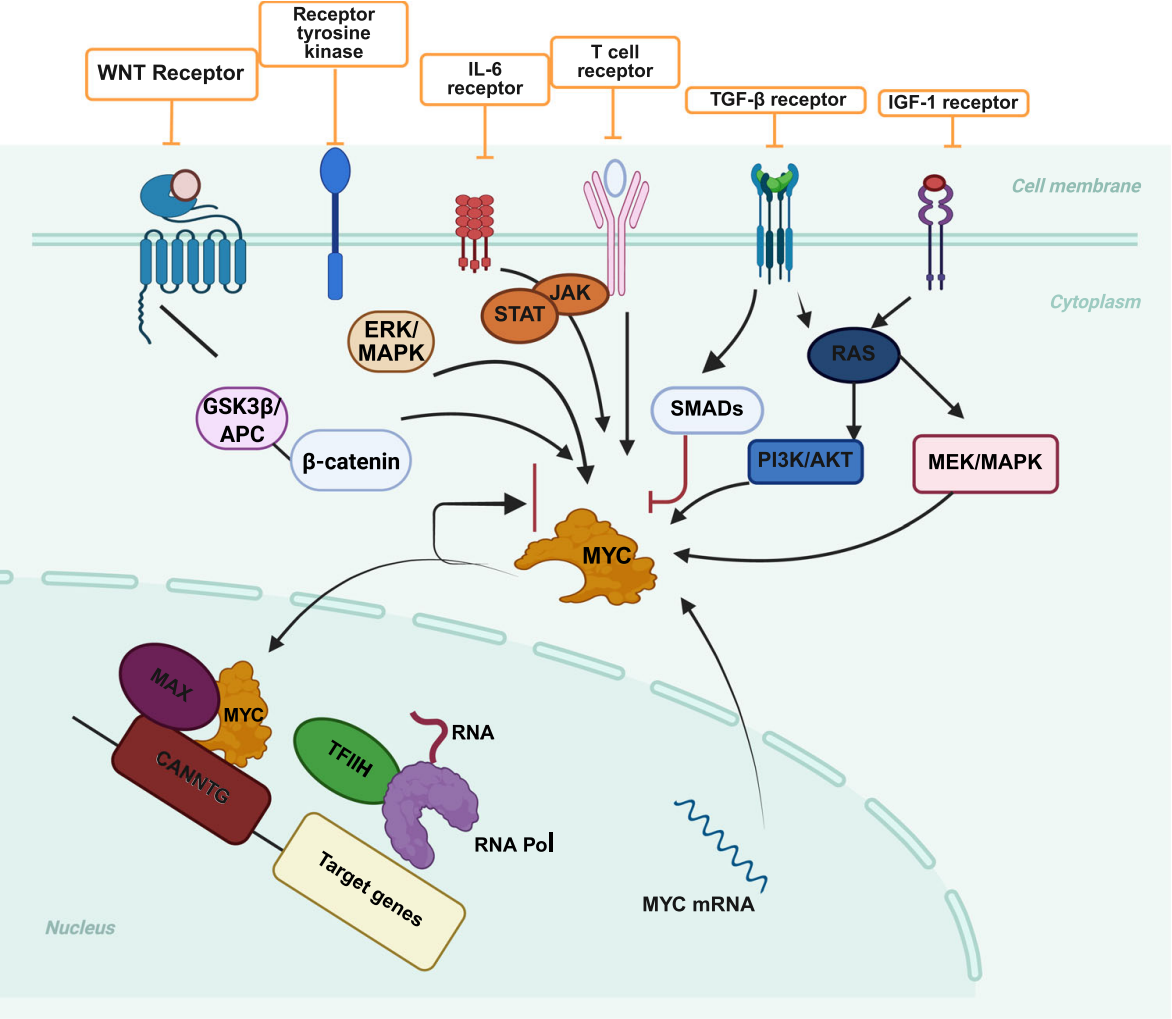
Schematic of the signaling landscape driving MYC expression and activity in cancer‐associated fibroblasts. Wingless‐related integration site (Wnt), interleukin‐6 (IL‐6), transforming growth factor‐beta (TGF‐β), and insulin‐like growth factor‐1 (IGF‐1), alongside receptor tyrosine kinase (RTK) and T cell receptor (TCR) engagement, propagate through β‐catenin, Janus kinase‐signal transducer and activator of transcription (JAK‐STAT), SMAD family member (SMAD), phosphoinositide 3‐kinase‐protein kinase B (PI3K‐AKT), and MAPK cascades to upregulate MYC. Accumulated MYC then dimerizes with MYC‐associated factor X (MAX), binds E‐box motifs (CACGTG), and recruits the basal transcription machinery, transcription factor II H (TFIIH), and RNA polymerase II (RNA pol II) to activate target genes governing extracellular matrix (ECM) production and cancer‐associated fibroblast (CAF) effector functions.

Cancer‐associated fibroblasts (CAFs) drive tumor invasion, immunosuppression, and therapy resistance through extracellular matrix (ECM) remodeling [88]. MYC, particularly c‐MYC, exerts comprehensive control over fibroblast phenotypic programming (Figure 3) [89, 90, 91, 92, 93]. Rather than merely responding to inflammation, fibroblasts actively convert these signals, like TGF‐β, IL‐1β, and oxidative stress into a c‐MYC dependent transcriptional program that remodels the matrix and consolidates an activated, myofibroblastic state [94]. c‐MYC‐activated CAFs assemble a stiff, collagen‐dense matrix that increases interstitial rigidity and licenses tumor cell motility. Concurrently, they secrete IL‐6 and CXCL12 to expand the regulatory T cell pool [95, 96]. However, c‐MYC's relative importance versus other CAF activators (e.g., YAP/TAZ, STAT3) remains poorly defined, and whether it is required for CAF maintenance or only initiation is unclear [97, 98]. N‐MYC indirectly promotes fibroblast activation via tumor‐derived SHH and PDGF signaling, while L‐MYC's role remains undefined [99, 100].

### 3.4 Nutrient Competition and Cellular Fitness

The TME is characterized by resource scarcity and spatial heterogeneity, where malignant and immune cells engage in metabolic competition [101]. MYC orchestrates this ecological dynamic by coupling nutrient acquisition to proliferation, indirectly suppressing immunity through metabolic exclusion [102]. c‐MYC upregulates glucose and amino acid transporters like SLC2A1, GLUT1, SLC1A5 and glycolytic enzymes, enabling high‐flux aerobic glycolysis and glutaminolysis [103, 104]. This creates a competitive disadvantage for tumor‐infiltrating lymphocytes dependent on the same nutrients. These programs are functionally interconnected with the comprehensive metabolic rewiring detailed in Section 5. Here we emphasize the competitive ecological consequences rather than enzymatic mechanisms. N‐MYC‐amplified tumors exhibit extreme glutamine dependence and suppress oxidative phosphorylation, impairing dendritic cell maturation. L‐MYC may coordinate with ASCL1 to shape neuroendocrine metabolic profiles, though direct evidence for its role in nutrient competition remains limited [105, 106]. Whether MYC‐driven metabolic competition represents adaptation to TME nutrient limitation or an intrinsic property of MYC‐transformed cells remains unresolved, a distinction with important therapeutic implications for restoring immune metabolic fitness.

## 4 Reciprocal Regulation Between MYC Family Members and ncRNAs

Building on the metabolic and microenvironmental regulatory roles of MYC described above, this section focuses on the reciprocal interactions between MYC family members and ncRNAs, highlighting how these multilayered regulatory circuits fine‐tune oncogenic outputs in a context‐dependent manner. Recent studies have illuminated a highly dynamic and reciprocal relationship between MYC and ncRNAs, including microRNAs (miRNAs), long ncRNAs (lncRNAs), and circular RNAs (circRNAs) [106]. Rather than being merely transcriptional byproducts, ncRNAs are now recognized as critical regulators of gene expression, epigenetic landscape, and protein stability. MYC functions not only as a master regulator of ncRNA expression but also as a target of ncRNA‐mediated control, establishing multilayered feedback and feedforward regulatory loops that modulate oncogenic output in a context‐specific manner [107].

### 4.1 Bidirectional Regulation Between MYC and miRNAs

MYC activates multiple oncogenic miRNA clusters that reinforce its own proliferative programs. Chief among these is the miR‐17∼92 cluster (oncomiR‐1), a polycistronic locus at 13q31.3 encoding six mature miRNAs: miR‐17, miR‐18a, miR‐19a, miR‐19b‐1, miR‐20a, and miR‐92a‐1 [108, 109]. c‐MYC occupies the miR‐17∼92 promoter to drive transcription of the polycistronic cluster. Individual miRNAs within this locus then dismantle specific tumor suppressive barriers: miR‐19a/b dampens PTEN, unleashing PI3K/AKT hyperactivation; miR‐17 and miR‐20a jointly repress CDKN1A (p21) and BCL2L11 (BIM), licensing cell cycle entry and disabling apoptosis; and miR‐18a destabilizes TGFBR2 and SMAD4, blunting cytostatic TGF‐β signaling [110, 111, 112, 113, 114, 115]. Beyond this cluster, c‐MYC transactivates miR‐9, which targets E‐cadherin (CDH1) to promote EMT, and miR‐221/222, which suppress CDKN1B (p27) and CDKN1C (p57) to accelerate G1/S transition [116, 117]. These miRNAs form positive feedback loops: miR‐19 indirectly stabilizes MYC by suppressing PTEN, sustaining MYC activity through AKT‐mediated inhibition of GSK3β‐dependent MYC degradation.

Conversely, tumor‐suppressive miRNAs directly target MYC family members posttranscriptionally, forming negative feedback loops frequently disrupted in cancer. The let‐7 family is extensively characterized: let‐7a, let‐7b, let‐7e, let‐7g, and let‐7i harbor seed sequences complementary to c‐MYC 3’UTR, and their ectopic expression downregulates MYC protein and suppresses proliferation. LIN28A/B binds pri‐let‐7 and blocks Drosha/Dicer processing; importantly, LIN28 is itself a let‐7 target, establishing the LIN28/MYC/let‐7 double‐negative axis, wherein MYC‐induced LIN28 suppresses let‐7 maturation, relieving MYC from repression. The miR‐34 family represents another critical axis: miR‐34a/b/c are directly transactivated by p53 and target c‐MYC 3’UTR [118, 119]. This p53/miR‐34/MYC axis means functional p53 suppresses MYC, whereas p53 loss eliminates this brake. Additional MYC‐targeting miRNAs include miR‐145 (silenced by promoter hypermethylation in colorectal and prostate cancers), miR‐193b (restoration suppresses proliferation in melanoma and hepatocellular carcinoma), and miR‐138 (targeting the coding region of MYC mRNA to circumvent 3’UTR‐shortening‐mediated escape) [120]. These tumor‐suppressive miRNAs are commonly downregulated in MYC‐driven cancers through transcriptional repression, epigenetic silencing, or defects in miRNA processing machinery.

Each MYC paralog exhibits distinct miRNA interaction profiles reflecting tissue‐specific oncogenic contexts. c‐MYC is the most extensively studied, with the broadest repertoire of validated interactions. In neuroblastoma, N‐MYC mirrors this logic: N‐MYC amplification drives overexpression of miR‐17∼92 and related paralogs (miR‐106b∼25, miR‐106a∼363), which target DKK3 and PTEN to sustain proliferative signaling [121]. Conversely, tumor‐suppressive miR‐542‐5p and miR‐204 directly target N‐MYC 3’UTR; their downregulation is associated with high‐risk neuroblastoma and poor clinical outcome. L‐MYC remains the least characterized, although studies in SCLC suggest it may intersect with neuroendocrine‐enriched miR‐375 and miR‐7, which regulate MYC network components in pulmonary neuroendocrine cells [122, 123].

The miR‐34a mimic MRX34 reached Phase I as the first miRNA cancer therapeutic (NCT01829971), producing partial responses and dose‐dependent target gene modulation in HCC and melanoma. Severe immune‐mediated toxicities nonetheless halted the trial, exposing the lipid carrier, not the miRNA payload itself, as the critical liability. This clinical failure has since driven intense investment in next‐generation delivery systems with improved tumor specificity and reduced immunogenicity. In parallel, antisense oligonucleotides (ASOs) directed against oncogenic miR‐17∼92 members are advancing through preclinical evaluation to restore tumor suppressor activity in MYC‐driven malignancies [124, 125]. Circulating levels of MYC‐regulated miRNAs (e.g., miR‐17∼92 family members and let‐7) show promise as noninvasive biomarkers for disease monitoring and therapeutic response prediction in MYC‐driven malignancies.

### 4.2 Paralog‐Specific LncRNA Regulatory Axes Within the MYC Family

While the functional convergence of c‐MYC, N‐MYC, and L‐MYC as transcriptional amplifiers is well established, emerging evidence highlights their distinct interactions with lncRNAs, influenced by cell lineage, developmental stage, and chromatin architecture [126]. These paralog‐specific lncRNA circuits not only underscore oncogenic heterogeneity but also provide potential therapeutic targets tailored to specific MYC‐driven tumor subtypes.

C‐MYC is the most extensively characterized paralog in this context. LncRNAs such as PVT1, CCAT1, and PCAT1 enhance c‐MYC transcriptional output through diverse mechanisms including protein stabilization, chromatin looping, and recruitment of epigenetic co‐activators [127, 128, 129]. In cancers, these lncRNAs are frequently overexpressed or co‐amplified with MYC, creating robust regulatory circuits that drive tumor progression. Notably, PVT1 has emerged as a critical dependency in c‐MYC‐amplified cancers. Preclinical studies have demonstrated that ASOs targeting PVT1 lead to MYC protein destabilization and tumor regression. Ongoing development of small molecules disrupting the PVT1–MYC complex represents a promising therapeutic frontier [130].

In N‐MYC‐driven tumors such as neuroblastoma, lncRNA‐mediated regulation is tightly integrated into developmental and metabolic programs. LncRNAs including N‐MYCOS, lncNB1, and NBAT1 regulate N‐MYC through mechanisms such as RNA duplex formation, epigenetic modulation, and enhancer activation. For example, lncNB1 enhances N‐MYC‐dependent glycolytic gene expression by recruiting WDR5 and H3K4 methyltransferases [131]. Therapeutic strategies here include siRNA‐loaded lipid nanoparticles and CRISPR interference (CRISPRi) targeting N‐MYC‐specific lncRNAs, which have shown efficacy in preclinical neuroblastoma models. Additionally, epigenetic inhibitors such as BRD4 or LSD1 antagonists may disrupt lncRNA‐facilitated enhancer landscapes permissive to N‐MYC expression [132].

L‐MYC, although less studied, may harbor similarly actionable lncRNA interactions. Given its role in SCLC, a malignancy with profound resistance to conventional therapies, characterizing L‐MYC‐associated lncRNAs may unlock new therapeutic approaches [133]. For instance, neuroendocrine lncRNAs such as NEAT1 and MIAT, which scaffold nuclear bodies and regulate splicing, are overexpressed in SCLC and could potentially intersect with L‐MYC‐driven transcription [41]. For instance, neuroendocrine lncRNAs such as NEAT1 and MIAT, which scaffold nuclear bodies and regulate splicing, are overexpressed in SCLC and could potentially intersect with L‐MYC‐driven transcription. Targeting these transcripts with RNA‐targeted CRISPR systems or locked nucleic acid (LNA) gapmers may attenuate L‐MYC‐dependent oncogenic programs [134].

Paralog‐specific targeting of MYC‐associated lncRNAs could circumvent the compensatory upregulation often observed with pan‐MYC inhibition. For example, inhibiting lncNB1 in N‐MYC‐high neuroblastoma may avoid induction of c‐MYC or L‐MYC, which limits monotherapy efficacy. Such targeted strategies may also minimize off‐target effects in normal proliferative tissues where only specific MYC paralogs are expressed. Furthermore, combination approaches integrating lncRNA‐targeted therapy with immune checkpoint blockade or metabolic inhibitors are gaining interest, as lncRNAs increasingly emerge as upstream regulators of immune evasion and biosynthetic control in MYC‐driven cancers [135, 136].

### 4.3 CircRNAs and Emerging Layers of MYC Regulation

In addition to miRNAs and lncRNAs, circRNAs are increasingly recognized as pivotal regulators within the MYC‐centered oncogenic network. CircRNAs, characterized by covalently closed loop structures that confer resistance to exonuclease‐mediated degradation, serve as stable posttranscriptional regulators, miRNA sponges, protein scaffolds, and even potential templates for small peptide translation. These unique properties position circRNAs as durable modulators of transcriptional programs driven by the MYC family [137].

Several circRNAs have been implicated in sustaining MYC signaling through diverse mechanisms. CircPVT1, derived from the same genomic locus as the MYC‐stabilizing lncRNA PVT1, is among the best‐characterized examples. It functions primarily by sponging tumor‐suppressive miRNAs such as miR‐125b, miR‐145, and miR‐30a, which target c‐MYC mRNA or its downstream effectors [138]. In gastric and colorectal cancers, circPVT1 overexpression correlates with MYC hyperactivity, enhanced proliferation, and poor prognosis [139]. Beyond miRNA sponging, circPVT1 stabilizes MYC protein by modulating ubiquitin ligase recruitment, suggesting a dual role at both RNA and protein levels. Other MYC‐associated circRNAs include circBA9.3 in leukemia and circFOXK2 in pancreatic cancer, which enhance MYC transcriptional output by recruiting RNA‐binding proteins or chromatin remodelers [140]. In neuroblastoma, circRNAs such as circDGKB and circKIF2C are selectively enriched in N‐MYC‐amplified tumors and may participate in lineage‐restricted regulation of N‐MYC stability, although mechanistic clarity is still emerging [45].

Given their inherent stability and tissue specificity, circRNAs represent attractive candidates for therapeutic targeting in MYC‐driven malignancies [141]. Preclinical efforts have employed RNA interference (RNAi), CRISPR‐Cas13‐based knockdown, and ASO technologies to silence pathogenic circRNAs such as circPVT1, leading to reduced MYC expression and impaired tumor growth [142]. Recent innovations in back‐splice junction‐specific targeting, using LNA gapmers or RNA‐guided Cas systems, have significantly improved the precision and efficiency of circRNA degradation [143]. Furthermore, circRNA vaccines are under early‐phase development, leveraging tumor‐enriched circRNAs as neoantigens to potentiate immune responses, particularly in MYC‐deregulated tumors with low mutational burden [42].

From a diagnostic standpoint, the abundance and stability of MYC‐related circRNAs in plasma or exosomes render them promising noninvasive biomarkers for disease monitoring and therapeutic response prediction [144]. Elevated levels of circPVT1 and circCCDC66 in circulating exosomes have been linked to MYC activity in colorectal and lung cancers, respectively, offering a real‐time window into MYC dynamics without requiring tumor biopsy [142].

Collectively, these findings position ncRNAs as integral components of the MYC regulatory network, operating across transcriptional, posttranscriptional, and epigenetic layers. This multilayered regulation not only reinforces MYC‐driven oncogenic programs but also provides additional nodes for therapeutic intervention. Notably, many of these regulatory effects converge on metabolic and transcriptional control, underscoring the integration of MYC‐driven gene regulation with cellular metabolic states [145].

## 5 MYC‐Mediated Metabolic Reprogramming and Structural Mechanisms of Transcriptional Control

Beyond its role as a transcriptional amplifier, MYC executes selective control over metabolic pathways by engaging chromatin‐specific enhancers and interacting with co‐regulators to drive cancer‐specific metabolic phenotypes [63, 141]. This section provides an overview of MYC's dual role in modulating core metabolic circuits and the structural underpinnings of its transcriptional selectivity, with detailed discussion of metabolic competition in the TME provided in Section 3.4.

### 5.1 Metabolic Rewiring Under MYC Control: Coordination of Biosynthetic and Bioenergetic Demands

MYC family transcription factors function as central orchestrators of metabolic reprogramming in cancer, enabling malignant cells to fulfill elevated anabolic and energetic demands. MYC, N‐MYC, and L‐MYC share conserved bHLH‐LZ domains that enable heterodimerization with MAX and binding to E‐box elements across the genome [1, 146]. Consequently, they regulate overlapping gene repertoires involved in glycolysis, glutamine metabolism, and lipid biosynthesis. Rather than enumerating individual enzymes and transporters (see Section 3.4 for detailed discussion), the core principle is that MYC integrates nutrient uptake, macromolecule biosynthesis, and redox balance to sustain tumor growth.

**FIGURE 4:**
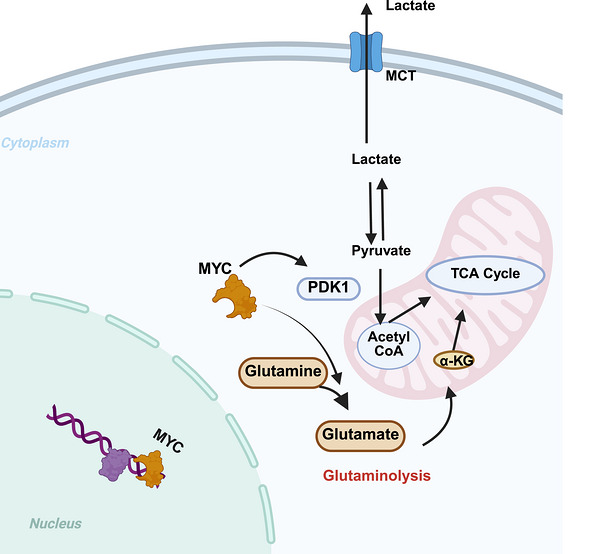
The key metabolic pathways influenced by MYC. MYC‐driven metabolic reprogramming in tumor cells. Nuclear MYC transcriptionally upregulates pyruvate dehydrogenase kinase 1 (PDK1), diverting pyruvate away from the tricarboxylic acid (TCA) cycle toward lactate production and export via monocarboxylate transporter (MCT). Simultaneously, MYC promotes glutaminolysis, which converts glutamine to glutamate and subsequently into alpha‐ketoglutarate (α‐KG) to replenish the intermediates of the TCA cycle. This concurrent activation of glycolytic flux and glutamine anaplerosis enables rapid adenosine triphosphate (ATP) generation and biosynthetic precursor supply for proliferating cancer cells.

MYC‐mediated metabolic reprogramming encompasses several interconnected hallmarks (Figure 4). First, MYC drives aerobic glycolysis by upregulating glycolytic enzymes and glucose transporters, promoting glucose catabolism even under normoxic conditions and supplying intermediates for biosynthesis [147]. Second, MYC renders many cancer cells glutamine‐addicted by activating glutamine transporters and glutaminase, facilitating anaplerotic flux into the TCA cycle. Third, MYC enhances serine‐glycine‐one‐carbon (SGOC) pathway flux to fuel nucleotide production and methylation capacity. Fourth, MYC promotes de novo lipogenesis and lipid uptake to support membrane biogenesis. Finally, MYC‐driven pentose phosphate pathway flux generates NADPH and glutathione to protect cells from oxidative stress. These programs are highly context‐dependent, shaped by MYC paralog identity, tissue of origin, and microenvironmental constraints [148, 149, 150]. Lipid metabolism is another pathway rewired by MYC to meet the demands of membrane biogenesis and cell signaling. Targeting MYC‐dependent metabolic vulnerabilities such as glutaminase inhibition, lactate transport blockade, or lipid synthesis disruption represents a promising therapeutic avenue [151, 152, 153].

### 5.2 Structural Basis of MYC Transcriptional Selectivity: Chromatin Engagement and Cofactor Interactions

Despite broad chromatin accessibility and capacity to bind canonical E‐box motifs (CACGTG), MYC family members exhibit remarkable transcriptional selectivity, targeting distinct gene subsets across cellular contexts [154]. This specificity arises from complex interplay between MYC protein structure, chromatin state, epigenetic landscapes, and cofactor dynamics [49].

The conserved bHLH‐LZ domain mediates MYC–MAX heterodimerization and E‐box binding within accessible chromatin [155]. However, genome‐wide binding profiles differ among MYC paralogs, reflecting intrinsic differences in their N‐terminal TADs and context‐dependent recruitment to chromatin hubs [156]. The TAD engages cofactors and chromatin modifiers, including histone acetyltransferases (GCN5, TIP60, p300/CBP), Mediator components, and P‐TEFb, facilitating RNA polymerase II pause release and transcriptional amplification [21, 153, 157, 158]. MYC preferentially associates with high histone acetylation regions, DNase hypersensitive sites, and active enhancers, particularly super‐enhancers, functioning primarily as a transcriptional amplifier rather than a de novo activator [52]. Paralog‐specific differences are evident: N‐MYC shows enhanced binding to neuronal enhancers in neuroblastoma, whereas MYC in epithelial tumors targets cell cycle and biosynthesis regulatory regions [2]. L‐MYC activity in SCLC depends on co‐occupancy with lineage factors ASCL1 and NEUROD1. PTMs further refine selectivity; for example, Ser62 phosphorylation enhances chromatin binding, while Thr58 phosphorylation primes ubiquitin‐mediated degradation, constituting a dynamic regulatory switch [82, 159].

MYC also orchestrates 3D genome reorganization through enhancer invasion and transcriptional condensate formation [160]. In MYC‐amplified neuroblastoma, N‐MYC hijacks neuronal super‐enhancers to create de novo enhancer–promoter loops that maintain oncogenic programs. Notably, resistance to CDK7 inhibition emerges through MYC‐mediated upregulation of alternative Mediator components MED12 and cyclin C, enabling transcriptional persistence [161]. Therapeutically, disrupting MYC‐cofactor interactions through BRD4 inhibitors (e.g., JQ1), CDK7 inhibitors (e.g., THZ1), or MYC–MAX disruptors (e.g., OmoMYC) impairs MYC‐driven transcriptional programs. These structurally grounded vulnerabilities highlight promising avenues for therapeutic intervention [141, 162].

## 6 MYC in Therapeutic Resistance and Emerging Targeting Strategies

**FIGURE 5:**
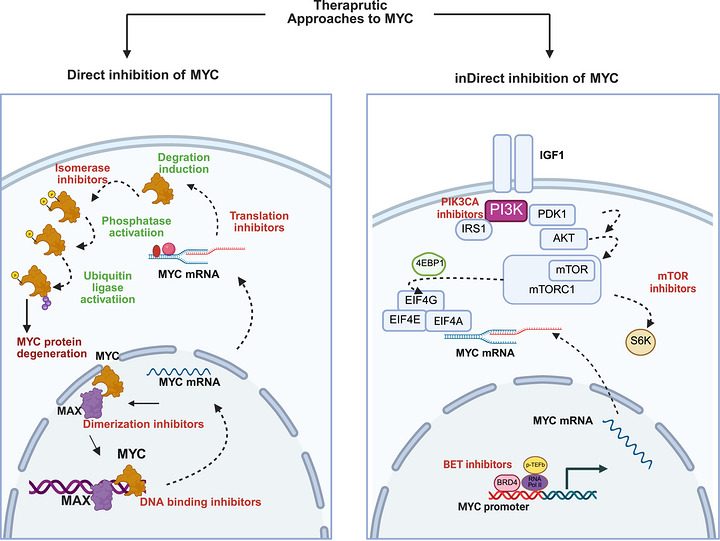
Classification of therapeutic strategies targeting MYC in cancer. Schematic classification of anti‐MYC interventions in cancer. Left panel, direct inhibitors attack MYC at multiple nodes: isomerase inhibitors, phosphatase activators, and ubiquitin ligase agonists drive MYC protein degradation; translation blockers suppress de novo MYC synthesis; dimerization and DNA‐binding inhibitors disrupt MYC–MAX assembly and chromatin engagement. Right panel, indirect strategies target MYC's regulatory network upstream phosphoinositide 3‐kinase (PI3K) pathway inhibitors, mechanistic target of rapamycin (mTOR) antagonists and bromodomain and extra‐terminal (BET) inhibitors each constrain MYC expression or activity through distinct signaling axes rather than engaging MYC itself.

Therapeutic resistance remains a central challenge in oncology, and MYC plays a multifaceted role in mediating both intrinsic and acquired resistance across various tumor types. As a pleiotropic regulator integrating transcriptional control, metabolic adaptation, and microenvironmental remodeling, MYC occupies a central node linking oncogenic signaling to therapy response [139] (Figure 5). Through its regulation of DNA repair, cell cycle checkpoints, metabolism, and the TME, MYC not only sustains tumor progression but also enables adaptation to chemotherapeutic, targeted, and immunologic interventions [163]. This section synthesizes recent advances in understanding MYC‐driven resistance mechanisms and highlights novel strategies aimed at directly or indirectly targeting MYC.

### 6.1 Mechanisms of MYC‐Mediated Therapeutic Resistance

MYC drives therapeutic resistance through several interconnected mechanisms. Overexpression of MYC suppresses proapoptotic pathways and upregulates antiapoptotic proteins, making tumor cells less susceptible to cell death induced by chemotherapy, targeted therapies, and immunotherapies [164]. MYC also promotes therapeutic resistance by enhancing metabolic flexibility, supporting increased biosynthesis and energy production, and by driving the expression of genes involved in cell cycle progression and DNA repair [45]. In specific malignancies, such as mantle cell lymphoma and melanoma, MYC activation is linked to resistance against CAR‐T therapy, kinase inhibitors, and immune checkpoint blockade, often mediated through upregulation of downstream effectors including HSP90 and CDK9 [140]. MYC mediates immune evasion by downregulating antigen presentation and suppressing T‐cell activation, and in some cancers, MYC‐driven epigenetic reprogramming maintains cancer stem cell phenotypes that contribute to tumor heterogeneity and relapse [165]. Context‐specific resistance paradigms illustrate the adaptive diversity of MYC‐driven tumors. MYC‐driven tumors exhibit compensatory metabolic switching upon glutaminase inhibition, with residual MYC activity upregulating glucose transporters and glycolytic enzymes to bypass glutamine dependency [166]. In EGFR‐mutant lung cancer, targeted therapy induces MYC‐dependent neuroendocrine transdifferentiation marked by ASCL1 activation and L‐MYC upregulation, representing a distinct resistance lineage requiring NOTCH pathway co‐inhibition [167]. Understanding these downstream effectors is essential for prioritizing therapeutic interventions.

### 6.2 Pharmacologic Inhibition of MYC: Direct and Indirect Approaches

Pharmacologic inhibition of MYC can be achieved through either direct or indirect approaches. Direct strategies focus on disrupting MYC's function or its interaction with essential partners, primarily by targeting MYC–MAX dimerization required for DNA binding. OmoMYC serves as the leading example, acting as a dominant‐negative inhibitor that blocks MYC activity and has shown efficacy in preclinical and early clinical studies with favorable toxicity profiles [139]. Alternative direct approaches (Table 2) include synthetic lethal targeting of MYC dependencies, MYC protein destabilization, and PROTAC‐mediated degradation [168]. RNA‐based therapeutics (siRNA, ASOs) have also been explored, though delivery efficiency and off‐target immune activation remain significant barriers [169]. While direct MYC inhibitors have historically faced challenges due to MYC's disordered structure and lack of enzymatic activity, recent advances, particularly cell‐penetrating peptide approaches like OmoMYC, are overcoming these barriers [11, 170]. However, fundamental constraints persist: MYC functions within a dynamic transcriptional network with extensive paralog redundancy (MYCN, MYCL), and adaptive transcriptional rewiring may restore oncogenic outputs even after MYC suppression [25]. (Table1)

**TABLE 2:** Therapeutic approaches to MYC and mechanisms.

Mode of action	Name	Mechanism	Evidence level
Prevents MYC dimerization	KJ‐Pyr‐9 [171]	Disrupts MYC–MAX dimerization, impairing DNA binding and transcriptional activation	Preclinical (in vivo evidence of xenograft tumor growth inhibition)
	L755507 [172]	Disrupts MYC–MAX dimerization, impairing DNA binding and transcriptional activation	Preclinical (in vitro only)
	MI3‐PD [173]	Disrupts MYC–MAX dimerization, impairing DNA binding and transcriptional activation	Preclinical (in vitro only)
	MYCMI‐6 [174]	Dominant‐negative inhibitor that sequesters MAX and blocks MYC‐dependent transcription	Preclinical (in vitro only)
	MYCro3 [175]	Dominant‐negative inhibitor that sequesters MAX and blocks MYC‐dependent transcription	Preclinical (in vitro only)
	OmoMYC [163]	Dominant‐negative inhibitor that sequesters MAX and blocks MYC‐dependent transcription	Preclinical + clinical (Phase I trial published)
	RASSF7 [176]	Destabilizes MYC by disrupting MYC–MAX complex formation	Preclinical (in vitro only)
	Omo‐103 [177]	Dominant‐negative inhibitor that sequesters MAX and blocks MYC‐dependent transcription	Preclinical + clinical (Phase I results reported)
	MYCi975 [59]	Dominant‐negative inhibitor that sequesters MAX and blocks MYC‐dependent transcription	Preclinical (in vitro only)
Protein inhibitors	6K465 [178]	Directly inhibits MYC protein function, impairing MYC‐dependent transcriptional activity	Preclinical (in vitro only)
	PLK1 [179, 180, 181]	Disrupts HSF1–MYC interaction, attenuating MYC‐mediated transcriptional programs	Preclinical (in vitro only)
	CPD.37 [182]	Disrupts MYC–MAX dimerization and inhibits E‐box‐dependent transcriptional activation	Preclinical (in vitro only)
	ME‐47 [183]	Interferes with MYC–MAX binding to E‐box elements, suppressing target gene transcription	Preclinical (in vitro only)
	GT19630 [184]	Induces proteasomal degradation of MYC and associated translational regulators	Preclinical (in vitro only)
	KSI‐3716 [185]	Inhibits MYC–MAX complex binding to target gene promoters	Preclinical (in vitro only)
	Curcumin [186]	Induces covalent modification of MYC, impairing its DNA‐binding and transcriptional activity	Preclinical (in vitro only)
Affects MYC transcription–related structures	IZCZ‐3 [187]	Stabilizes G‐quadruplex structures in MYC promoter, inhibiting transcription	Preclinical (in vitro only)
	m‐Se3 [188]	Stabilizes G‐quadruplex structures in MYC promoter, inhibiting transcription	Preclinical (in vitro only)
	QN‐1 [189]	Stabilizes G‐quadruplex structures in MYC promoter, inhibiting transcription	Preclinical (in vitro only)
	Thiazole peptide TH3 [190]	Stabilizes G‐quadruplex structures in MYC promoter, inhibiting transcription	Preclinical (in vitro only)

Direct MYC inhibition remains difficult, so targeting upstream and codependent pathways offers a practical alternative (Table 3). YAP/TAZ, downstream of the Hippo pathway, sustain oncogenic transcription by cooperating with TEAD to activate MYC target genes; verteporfin disrupts this interaction and shows preclinical activity, though selectivity issues have stalled clinical progress [191, 192, 193]. The p53 pathway provides another angle: MDM2 inhibitors such as idasanutlin and milademetan restore p53 function, which represses MYC transcription and promotes its degradation, partly through miR‐34a induction, yet p53 mutations in many tumors limit single‐agent efficacy [194, 195, 196, 197]. PI3K/AKT/mTOR signaling controls MYC translation via eIF4E and stabilizes MYC by inactivating GSK‐3β; inhibitors of this cascade suppress MYC in preclinical models, but feedback activation and toxicity narrow their use, especially in PTEN‐deficient cancers where MYC dependency is pronounced [198, 199]. WNT/β‐catenin drives MYC expression through TCF/LEF; porcupine inhibitors (LGK974), CBP/β‐catenin antagonists (ICG‐001), and TNKS inhibitors (NVP‐TNKS656) attenuate this axis but show modest activity as monotherapy in colorectal cancer [200, 201, 202, 203]. BRD4, CDK7, and CDK9 represent transcriptional codependencies: BET inhibitors evict BRD4 from MYC super‐enhancers, while CDK7/9 inhibitors stall transcriptional elongation, though off‐target effects and resistance through WNT/β‐catenin or BRD4 mutation argue for combination regimens such as BET plus PI3K or CDK7 blockade [128, 204, 205]. Taken together, these approaches suggest that durable MYC suppression will probably require concurrent pathway inhibition and careful patient selection based on molecular profiling.

**TABLE 3:** MYC‐targeting pathway: representative agents and clinical status.

Target pathway	Representative agents	Mechanism of MYC suppression	Clinical stage	Key indications
YAP/TAZ [12, 191, 192, 193, 206]	Verteporfin	Disrupts YAP–TEAD interaction; downregulates MYC target genes.	Preclinical/Phase I repurposing	HCC; NSCLC
p53/MDM2 [195]	Idasanutlin; milademetan	p53 activation induces MYC transcriptional repression and miR‐34‐mediated MYC degradation.	Phase I–II	AML; liposarcoma
PI3K/AKT/mTOR [199]	Alpelisib; ipatasertib; everolimus	Reduces eIF4E‐dependent MYC translation; enhances GSK‐3β‐mediated MYC degradation.	Approved (selected indications); Phase II–III combinations	ER+ breast cancer; HNSCC
PTEN pathway [43, 108]	Gene therapy approaches; PI3K combination regimens	PTEN restoration promotes MYC degradation via GSK‐3β.	Preclinical	Prostate; breast cancer (PTEN‐null)
WNT/β‐catenin [201, 207]	LGK974; ETC‐159;ICG‐001; NVP‐TNKS656	Inhibits TCF/LEF‐mediated MYC transcription.	Phase I–II	CRC (WNT‐mutant)
BRD4/BET [59, 71]	JQ1; OTX015; birabresib; pelabresib	Displaces BRD4 from MYC super‐enhancer; suppresses MYC transcription.	Phase I–II	Hematologic malignancies; NUT carcinoma
CDK7 [56, 69, 161, 205]	THZ1; SY‐1365; samuraciclib	Inhibits transcription initiation at MYC‐dependent genes.	Phase I–II	AML; solid tumors
CDK9 [140, 158, 204]	AZD4573; KB‐0742	Blocks P‐TEFb‐mediated pause release; suppresses MYC transcription.	Phase I	Hematologic malignancies

### 6.3 Preclinical Studies, Clinical Trials, and Critical Assessment

Several MYC‐targeted agents have progressed from preclinical characterization to early‐phase clinical trials, though translation to clinical benefit remains limited and highly context‐dependent [57]. Among direct inhibitors, OmoMYC and its clinical derivative OMO‐103 represent the most advanced candidates, demonstrating acceptable safety profiles and evidence of on‐target transcriptional modulation in Phase I studies involving patients with advanced solid tumors [177]. Preclinical models suggest that MYC inhibition induces tumor regression primarily through proliferative arrest and differentiation rather than apoptosis, with implications for response durability. MYC inhibition synergizes with PARP inhibitors in triple‐negative breast cancer by exacerbating replication stress, though clinical validation is pending [163, 208].

Indirect approaches have shown mixed results. BET inhibitors including birabresib and pelabresib demonstrated modest tumor growth suppression in early‐phase trials, with responses generally transient due to compensatory transcriptional programs and incomplete MYC suppression [209, 210]. PIM kinase inhibition with AZD1208 showed preclinical efficacy but limited clinical benefit. CDK7/9 inhibitors have shown early signals of activity in MYC‐high hematologic malignancies, though their broad transcriptional effects complicate interpretation of mechanism‐specific efficacy [1]. Critical assessment reveals persistent gaps between preclinical efficacy and clinical outcomes. Single‐agent BET inhibition has failed to produce durable responses in most solid tumors, likely reflecting compensatory WNT/β‐catenin activation and alternative enhancer engagement. Similarly, MDM2 inhibitors achieved limited single‐agent activity in p53‐wildtype tumors, with acquired resistance frequently involving p53 mutation or downstream pathway reactivation. PI3K/AKT/mTOR inhibitors have demonstrated clinical benefit primarily in molecularly selected populations (e.g., PIK3CA‐mutant breast cancer), underscoring the need for predictive biomarkers. The translational gap likely reflects context‐specific MYC dependency, functional paralog redundancy, and adaptive transcriptional rewiring capacity [211].

### 6.4 Overcoming Resistance: Combination Therapies and Precision Targeting

Combination strategies integrating MYC‐directed therapies with existing modalities are emerging as rational approaches to overcome resistance [212]. Coadministration of BET inhibitors or OmoMYC with immune checkpoint blockade demonstrated synergistic tumor clearance in MYC‐overexpressing models, highlighting MYC‐immunotherapy combinations as promising approaches [213]. However, these effects remain largely restricted to experimental settings, and translational feasibility requires further validation [214]. Targeting MYC alongside metabolic pathways such as glycolysis or glutaminolysis can prevent metabolic compensation [215, 216]. MYC‐driven tumors exhibit heightened sensitivity to DNA damage when co‐treated with PARP or POLQ inhibitors, suggesting that MYC–DNA repair inhibition combinations may sensitize resistant cells [217]. In EGFR‐mutant cancers, MYC expression levels predict osimertinib efficacy, and MYC knockdown restores drug sensitivity in resistant cell lines. Nevertheless, concerns regarding specificity and off‐target effects warrant careful evaluation.

Dual metabolic inhibition of glycolysis and glutaminolysis, combined with MYC‐targeted agents, represents a compelling strategy to overcome resistance. In MYC‐expressing tumors, pharmacological glycolysis inhibition reduces lactate production and oxygen consumption, while glutamine metabolism disruption deprives cancer cells of critical carbon and nitrogen sources. Despite promising preclinical results, compensatory metabolic rewiring and systemic toxicity may limit therapeutic windows [218]. Overall, while these paradigms underscore the rationale for precision targeting, substantial challenges remain, including tumor heterogeneity and the translational gap between preclinical efficacy and patient outcomes.

## 7 Intratumoral Microbiota: A Microbial Dimension in MYC‐Driven Oncogenesis and Therapeutic Evasion

Emerging evidence positions the intratumoral microbiota as a critical exogenous regulatory layer intersecting with MYC oncogenic networks. Specific bacterial communities actively modulate MYC activation, metabolic reprogramming, and therapeutic responses through direct host–pathogen interactions and systemic effects. This section delineates the mechanistic crosstalk between intratumoral microbiota and MYC signaling [219].

### 7.1 Microbial Activation of MYC via the Wnt/β‐Catenin Nexus

A conserved paradigm involves bacterial virulence factors hijacking Wnt/β‐catenin signaling to drive MYC transcription. In colorectal cancer, *Fusobacterium nucleatum* exploits its FadA adhesin to engage E‐cadherin, triggering β‐catenin nuclear translocation and TCF/LEF‐dependent MYC upregulation. Enterotoxigenic *Bacteroides fragilis* secretes the metalloprotease toxin BFT, which cleaves E‐cadherin to initiate β‐catenin stabilization and MYC induction. Beyond the gut, *Helicobacter pylori* deploys the CagA effector and *Salmonella* utilizes AvrA to perturb the E‐cadherin/β‐catenin axis, converging on MYC activation [220, 221]. These findings establish a “microbe–adhesin–E‐cadherin–β‐catenin–MYC” axis as a shared mechanism whereby intratumoral bacteria circumvent host regulatory checkpoints.

### 7.2 Microbiota‐Driven Metabolic Reprogramming and Immune Evasion

Intratumoral microbiota profoundly influence the metabolic landscape of MYC‐driven tumors. Microbial‐derived secondary bile acids activate β‐catenin signaling, upregulating MYC expression and triggering endoplasmic reticulum stress responses that paradoxically support tumor survival. MYC‐driven tumors with high bacterial loads demonstrate enhanced glutaminolysis and glycolytic flux, depleting essential nutrients required for immune effector function and reinforcing immune evasion [222, 223].

Moreover, microbial metabolites modulate the competitive metabolic dynamics within the tumor ecosystem. MYC‐driven tumors exhibiting high intratumoral bacterial load demonstrate enhanced glutaminolysis and glycolytic flux, creating a metabolically hostile environment that depletes essential nutrients (glucose, tryptophan, arginine) required for immune effector function [224]. This metabolic reprogramming not only fuels MYC‐dependent biosynthetic demands but also reinforces immune evasion, establishing a symbiotic relationship between microbial colonization and MYC‐mediated metabolic fitness [225].

### 7.3 Therapeutic Implications: Targeting Microbiota to Modulate MYC

The recognition of microbiota as MYC modulators opens innovative therapeutic avenues. Preclinical models demonstrate that targeted eradication of intratumoral *F. nucleatum* using liposomal silver–metronidazole formulations promotes CD8+ T‐cell infiltration. Engineered probiotics (e.g., *E. coli* Nissle 1917 derivatives) can reverse M2‐to‐M1 macrophage polarization, disrupting the MYC‐dependent immunosuppressive ecosystem. These interventions may synergize with direct or indirect MYC inhibitors by alleviating compensatory resistance mechanisms [226]. Antibiotic‐mediated microbiome modulation has shown promise in enhancing immune checkpoint blockade efficacy, though preservation of microbiome diversity remains essential to prevent adverse outcomes.

## 8 Conclusion

MYC functions as a nodal amplifier of oncogenesis, integrating proliferative, metabolic, and immunomodulatory signals across diverse tumor contexts [227, 228]. Unlike classical oncogenes driven by mutation, MYC exerts its oncogenic effects primarily through overexpression, acting as a dosage‐sensitive regulator of anabolic growth and transcriptional output [229, 230]. MYC promotes cell cycle progression via cyclin‐dependent kinase activation while simultaneously reprogramming metabolism including glycolysis, glutaminolysis, and lipogenesis, to sustain biosynthesis and redox balance [231, 232]. These changes extend beyond intrinsic tumor cell fitness, as MYC reshapes the TME by recruiting immunosuppressive cells, modulating vasculature, and activating CAFs. Importantly, MYC inhibition in preclinical models induces reversible tumor regression, often via cell quiescence or differentiation rather than apoptosis, and with minimal toxicity to normal tissues, supporting a favorable therapeutic window for intervention [233, 234, 235].

Despite its central role, MYC remains challenging to target due to its intrinsically disordered structure, lack of enzymatic activity, and essential physiological functions in normal proliferative tissues. Recent advances including dominant‐negative constructs (OmoMYC/OMO‐103), PROTAC‐mediated degradation strategies, and small‐molecule inhibitors of the MYC–MAX interface highlight the emerging feasibility of direct targeting. Complementary indirect approaches targeting BRD4/BET, CDK7/9, PI3K/AKT/mTOR, WNT/β‐catenin, p53/MDM2, and YAP/TAZ pathways expand the therapeutic toolkit by suppressing MYC at transcriptional, translational, and posttranslational levels [236, 237, 238]. Nevertheless, many indirect agents exert broad effects on global transcriptional programs, raising concerns about mechanism specificity and on‐target toxicities that have limited single‐agent clinical efficacy across unselected patient populations.

Critical unresolved challenges must be addressed to advance the field. First, functional redundancy among MYC paralogs (MYC, N‐MYC, and L‐MYC) enables compensatory activation that limits durability of single‐agent therapies. Second, MYC‐driven tumors exhibit substantial transcriptional and metabolic plasticity, allowing rapid adaptation through enhancer reprogramming and alternative pathway activation [239]. Third, the translational gap between robust preclinical efficacy and modest clinical outcomes persists, reflecting context‐specific MYC dependency, inadequate patient stratification biomarkers, and incomplete understanding of which MYC‐overexpressing tumors are truly MYC‐addicted [240, 241]. Fourth, MYC's dual role in tumor immunity, simultaneously promoting immune evasion via PD‐L1 upregulation while modulating the antigen presentation machinery, complicates its integration with immunotherapy [9]. Fifth, the emerging role of intratumoral microbiota as both facilitators and modulators of MYC signaling adds another dimension of complexity to therapeutic resistance and response prediction.

Future strategies must prioritize rational combination regimens tailored to specific molecular contexts, such as BET plus CDK7 inhibitors in hematologic malignancies, PI3K plus MYC blockade in PTEN‐deficient solid tumors, or MDM2 plus BET inhibition in p53‐wildtype cancers. Moreover, they should develop predictive biomarkers to identify truly MYC‐addicted tumors [242, 243]. Integrating single‐cell and spatial multi‐omics approaches will be essential to define context‐specific vulnerabilities, track clonal adaptation, and map resistance trajectories in real time. The convergence of microbiome science with MYC biology further suggests that microbe‐targeted interventions, including engineered probiotics and targeted antimicrobial strategies, may sensitize resistant tumors and enhance immunotherapy responses. Looking forward, the development of paralog‐selective inhibitors, cell‐penetrating peptide delivery systems with improved tumor targeting, and MYC‐specific PROTACs with optimized E3 ligase recruiters will be critical priorities [22, 28, 32, 37]. Ultimately, shifting from a gene‐centric view to a network‐aware, context‐adapted framework one that systematically incorporates paralog dynamics, microenvironmental crosstalk, metabolic dependencies, transcriptional codependencies, and microbial interactions may convert MYC from an elusive target into a transformative precision oncology opportunity.

## Author Contributions

H.Y. conceived the review and established the intellectual framework. N.Z. led literature analysis, designed all figures and tables, and drafted the manuscript. L.W. performed systematic literature screening on metabolic reprogramming and noncoding RNA regulation, and managed references. C.T. analyzed tumor microenvironment and therapeutic resistance literature, and verified clinical trial data. P.L. designed and refined all schematic illustrations and optimized graphical presentation. W.Z. critically reviewed therapeutic targeting sections, provided pharmacological expertise, and revised the manuscript for accuracy. All authors reviewed, edited, and approved the final version.

## Funding

The authors have nothing to report.

## Ethics Statement

The authors have nothing to report.

## Conflicts of Interest

The authors declare no conflicts of interest.

## Data Availability

This is a review article and no original data were generated.
